# Prevalence of gastroparesis symptoms and its associated factors among type 2 diabetes mellitus patients in West Bank in Palestine: a national cross-sectional study

**DOI:** 10.3389/fmed.2025.1499725

**Published:** 2025-02-12

**Authors:** Diya Asad, Qusai Zreqat, Shahd Idais, Bara'ah Hussein, Alaa Ayyad, Marah Hunjul, Hamzeh M. I. AbuGharbieh, Haroun Neiroukh, Areen Zuhour, Salsabeel AbuKhalaf, Nour Al-Atrash, Roa Alzughayyar, Yumna Njoum, Hussein Hallak

**Affiliations:** ^1^Faculty of Medicine, Al-Quds University, Jerusalem, Palestine; ^2^Medical Research Club, Al-Quds University, Jerusalem, Palestine; ^3^School of Medicine, Faculty of Medicine and Health Sciences, An-Najah National University, Nablus, Palestine; ^4^Palestinian Medical Complex, Ramallah, Palestine

**Keywords:** diabetes, diabetic gastroparesis, glycosylated hemoglobin, delayed gastric emptying, cross-sectional study, GCSI

## Abstract

**Introduction:**

Diabetic gastroparesis (DGP) is defined as delayed gastric emptying without any mechanical obstruction in diabetic patients.

**Methods:**

We conducted a cross-sectional study using an Arabic-validated translated version of the Gastroparesis Cardinal Symptom Index (GCSI). A total of 3,542 diabetic patients were interviewed, of whom 91.6% were finally included in the analysis.

**Results:**

DGP symptoms were present in 14.5% of the study population, of which 10.2% had a GCSI score of severe disease. Further analysis of individuals with GCSI scores≥1.9 (14.5%; 470) revealed that 50.8% of them visited a doctor at least once, and 18% had been hospitalized due to DGP symptoms. However, only nine patients (1.9%) were diagnosed with DGP. The most common symptoms were stomach fullness and early satiety. The binary regression model showed that DGP symptoms were more likely to occur in patients who had diabetes for >10 years and glycosylated hemoglobin >9. Furthermore, the model revealed that females were at a higher risk of developing DGP.

**Discussion:**

This was the first study in Palestine on DGP, which showed that the condition is underdiagnosed. This is not only because of the unavailability of standard diagnostic methods but also due to the under appreciation of gastrointestinal complaints in diabetic patients.

## Highlights

- The prevalence of diabetic gastroparesis (DGP) in Palestine has not been studied before.
- This study aimed to investigate the prevalence of DGP and its associated factors in type 2 diabetes mellitus patients.
- DGP was underdiagnosed. The most common symptoms were stomach fullness and early satiety. DGP symptoms were more likely to occur in patients who had diabetes for ≥10 years and glycosylated hemoglobin ≥9. Furthermore, females were at a higher risk of developing DGP.
- Diagnosis and management that translates into clinical practice healthcare should be provided to DGP patients. Physicians should pay close attention to patients manifesting risk factors.

## Introduction

Diabetic gastroparesis (DGP) is defined as a delay in gastric emptying without any mechanical obstruction in diabetic patients (1). Other potential causes of gastroparesis include abdominal or esophageal surgery, Parkinson’s disease, collagen vascular disorders, thyroid dysfunction, liver disease, chronic renal insufficiency, intestinal pseudo-obstruction, or idiopathic (2). DGP is associated with nausea, vomiting, early satiety, postprandial abdominal fullness, and discomfort. It affects patients with long-standing diabetes mellitus, which is often complicated by neuropathy, nephropathy, and retinopathy (1–3). In general, the severity of DGP increases with an increase in age, body mass index (BMI), and glycated hemoglobin (HbA1C) levels (4). Moreover, female patients have been reported to be at a higher risk of developing DGP in comparison to males (4). The underlying mechanism of this trend is not fully understood but is thought to be linked to estrogen hormone (4, 5). The prevalence of diabetes-associated gastrointestinal symptoms has been reported to range between 5 and 12%. DGP largely affects the quality of a patient’s life through hospitalizations, emergency department visits, or doctor visits, not to mention their financial costs (1, 6). It can also result in several complications, including esophagitis, Mallory-Weiss tears from chronic nausea and vomiting, malnutrition, volume depletion with acute renal failure, electrolyte disturbances, and bezoar formation (7–9).

Early diagnosis of DGP along with glucose control, dietary modifications, and the use of prokinetics, is required to mitigate the downside effects of the condition and achieve clinical improvement (5, 6). The golden standard for diagnosing DGP is gastric scintigraphy, which is an expensive procedure and is associated with a small but noticeable radiation risk (6). Therefore, it is rational to use a stepwise approach with a safer and more convenient screening method that selects patients according to the presence of cardinal symptoms suggestive of DGP before proceeding to gastric scintigraphy. Management of DGP should involve different aspects of optimization of glycemic control, correction of electrolyte imbalance, when necessary, dietary modifications, nutritional support, pharmacological therapy, and, when indicated in selected cases, surgical and endoscopic interventions (10). Such management would require a multidisciplinary team consisting of gastroenterologists, diabetologists, dieticians, and surgeons. Emerging evidence highlights the potential role of botanicals in managing Type 2 Diabetes Mellitus (T2DM) and their possible implications for diabetic complications, including gastroparesis. Botanicals such as mulberry and quercetin have demonstrated anti-inflammatory and glycemic control properties, which may indirectly influence the progression of complications like gastroparesis. Additionally, ginseng and Oolong tea have been explored as part of integrative approaches to improving metabolic health, potentially reducing the burden of gastrointestinal complications. These therapeutic approaches may complement conventional treatments and provide a broader framework for managing diabetes and its associated conditions (11–14).

The prevalence of DGP has not been previously investigated in Palestine. Therefore, the present national study aimed to: (i) determine the prevalence of DGP in the West Bank in Palestine, (ii) study the association between DGP and different variables including sex, duration of diabetes, and severity, and (iii) identify potential risk factors of the disease in a general non-clinical Palestinian population.

## Research design and methods

### Ethical considerations

This study was conducted in accordance with the tenets of the Declaration of Helsinki, after obtaining approval from the Research Ethics Committee of Al-Quds University. Informed consent was obtained from all the participants before the commencement of the study.

### Study design and population

This national cross-sectional study was conducted between February 2020 and January (15) at the West Bank of Palestine to assess the prevalence of DGP-related symptoms in 3542 individuals with type 2 diabetes mellitus.

### Sampling method and location

Participants were selected using a simple randomization approach. Trained data collectors (medical students) were instructed to approach eligible patients at random within the seven participating governmental hospitals. These hospitals were selected based on their capacity (≥100 beds) and geographic representation. To ensure randomness, data collectors were explicitly instructed not to follow any systematic pattern (e.g., approaching every second or third patient) but instead to select participants in a manner that provided every eligible individual with an equal chance of inclusion. This method ensured a representative sample and minimized selection bias.

Participants were selected from seven Palestinian governmental hospitals that met our criterion of more than 100 beds (16). The participants were distributed among the following governorates: Hebron, Ramallah, Bethlehem, Nablus, Tulkarem, and Jenin.

### Inclusion and exclusion criteria

Adult Palestinian patients aged ≥18 years with type 2 diabetes mellitus were invited to participate in this study. Participants with condition known to affect gastric motility, liver diseases, gastrointestinal diseases, previous surgeries, collagen vascular diseases, thyroid disorders, Parkinson’s disease, and chronic renal insufficiency, were excluded. Moreover, patients receiving medications that could alter gastric emptying, including opiates, anticholinergic, tetrahydrocannabinol (THC), and glucagon, were also excluded (1). Patients who refused to complete the survey were excluded from the study as well (2).

### Sample size

According to the Palestinian Central Bureau of Statistics (PCBS), the population of the West Bank of Palestine was estimated to approximately 2.99 million (17). The reported prevalence of diabetes in Palestine is high (15.3%) (18). Furthermore, the prevalence of gastroparesis-related symptoms in diabetic patients is estimated to range from 5 to 12% (18, 19). To determine the required sample size, we used a sample size calculator developed by the Australian Bureau of Statistics for simple random samples (15), assuming a 95% confidence level and 5% margin of error. Based on these parameters, the minimum sample size was 381. However, the final dataset included 470 patients with DGP, ensuring sufficient power for the study.

### Symptom assessment using the gastrointestinal cardinal symptom index (GCSI)

We used the GCSI, a subset of items from the longer Gastrointestinal Disorders-Symptom Severity Index (PAGI-SYM) instrument that was developed to evaluate the severity of symptoms related to gastroparesis (20). The GCSI is a validated and widely utilized patient-reported outcome measure designed to evaluate the severity of key gastroparesis symptoms, including nausea, bloating, and postprandial fullness. Foundational studies, have demonstrated its validity in terms of internal consistency, reliability, and sensitivity to symptom variations (2, 21)

Nonetheless, we acknowledge that the GCSI is not intended to serve as a definitive diagnostic modality for gastroparesis, as it does not provide direct measurement of gastric emptying. Gastric scintigraphy, recognized as the gold standard, remains essential for confirming delayed gastric emptying (6).

The GCSI consists of three subcategories of patient assessments of upper gastrointestinal disorders, including nausea/vomiting, post-eating/early satiety symptoms, and bloating. Generally, the GCSI includes nine questions, and each question is rated by a responder according to the severity of symptoms using a 5-point Likert scale ranging between 0 and 5 (wherein 0 refers to no symptoms, while 5 indicates severe symptoms). The average of all nine questions was considered the final score (20). The total score for GCSI patients was entered into a database, and values ≥1.9 were selected as indicative of gastroparesis symptoms, based on literature (21).

The rationale for employing the GCSI in this context lies in its practicality and accessibility, particularly in resource-limited settings like Palestine, where advanced diagnostic tools such as gastric scintigraphy are not readily available. By utilizing the GCSI, we sought to address a critical gap in symptom screening, facilitating the identification of patients who may benefit from further diagnostic evaluation and tailored management strategies. This aligns with the broader objective of the study, which is to raise awareness about the often-overlooked burden of gastrointestinal symptoms among diabetic patients.

### Translation of GCSI

The GCSI was not available in Arabic. Due to cultural and linguistic differences, we followed the linguistic validation process for translation as outlined in the Clinical Outcome Assessment (COA) provided by Mapi Research Trust (22). Initially, two bilingual translators with relevant healthcare-related translation experience translated the instrument from English to Arabic. This version was then back-translated into English, by two other bilingual translators. To ensure accuracy and cultural appropriateness of the translation, cognitive interviews were conducted with five native Arabic-speaking participants who were over 18 years of age and had diabetes. A pilot study was conducted with 52 respondents to test the clarity of survey questions, and their data were excluded from the analysis. Finally, the translated version underwent proofreading. The Internal consistency of the Arabic GCSI was evaluated using Cronbach’s alpha, which reached an acceptable value (*α* = 0.909).

### Survey description

Participants completed a four-part questionnaire. The first section collected demographics and lifestyle information including age, sex, weight, height, level of education, smoking status, fluid intake, and vitamin use. The second part gathered data on diabetes duration, most recent fasting blood sugar (FBS), and HbA1C levels, as well as information about diabetes control and management option. The third section was dedicated to screening using the Arabic version of the GCSI. The final section targeted participants with a GCSI score ≥ 1.9, focusing on diabetic gastroparesis (DGP). In this part, they were asked about the diagnostic status, initial symptom, most bothersome symptom, most frequent symptom, doctor and hospital visits, and a satisfaction Likert scale regarding the current management options they were adherent to.

### Data collection

Participants were invited for a face-to-face interview to complete the questionnaire, which took on average of approximately 10 min. Participants agreed verbally to an informed consent at the beginning of the interview. Data was collected using the “Kobo Toolbox,” a user-friendly online tool for building digital surveys and collecting high-quality data via smartphones (23). Data collection was done by trained medical students, who owned compatible devices and were instructed on how to use “Kobo Toolbox.

Additionally, they were also trained on how to approach participants, ensure continuous follow-up, and guide them through the completion of the questionnaire. To ensure data completeness, participants could not proceed to the next page until all required fields were filled out, ensuring that all necessary information was collected.

### Statistical analysis

Data entry and analysis were conducted using IBM Statistical Package for Social Studies (SPSS, Windows version 23). A statistician performed the analysis to ensure a blind assessment of the results. Continuous variables (age, duration of diabetes, FBS, height, weight, BMI, HbA1C, and GCSI scores) were summarized as mean ± SD. Categorical variables were summarized using frequency distributions and percentages. A GCSI score ≥ 1.9 was determined as having DGP, and a score of ≥3.5 was defined as a severe form of the disease. HbA1C was categorized into three subgroups (<7, 7–9, and > 9). Duration of diabetes was also categorized (<5 years, 5–10 years, >10 years). We used the Pearson Chi-square test and independent-sample t-test to assess characteristic differences between patients with and without DGP. Pearson Chi-square test was used to evaluate the correlation between GCSI score and HbA1C on one side and with diabetes duration on the other side. To compare patients with DGP and those without DGP, we used a post-hoc one-way ANOVA test to assess the correlation among GCSI score, duration of diabetes, and HbA1C categories. Binary logistic regression was conducted to study the probability of gastroparesis in diabetic patients using multiple variables (age, sex, HbA1C, duration of diabetes, and BMI). Furthermore, we constructed a binary logistic regression model to determine the factors affecting the severity of DGP-related symptoms. A *p*-value of ≤0.05 was considered significant for all tests.

## Results

**Figure 1:**
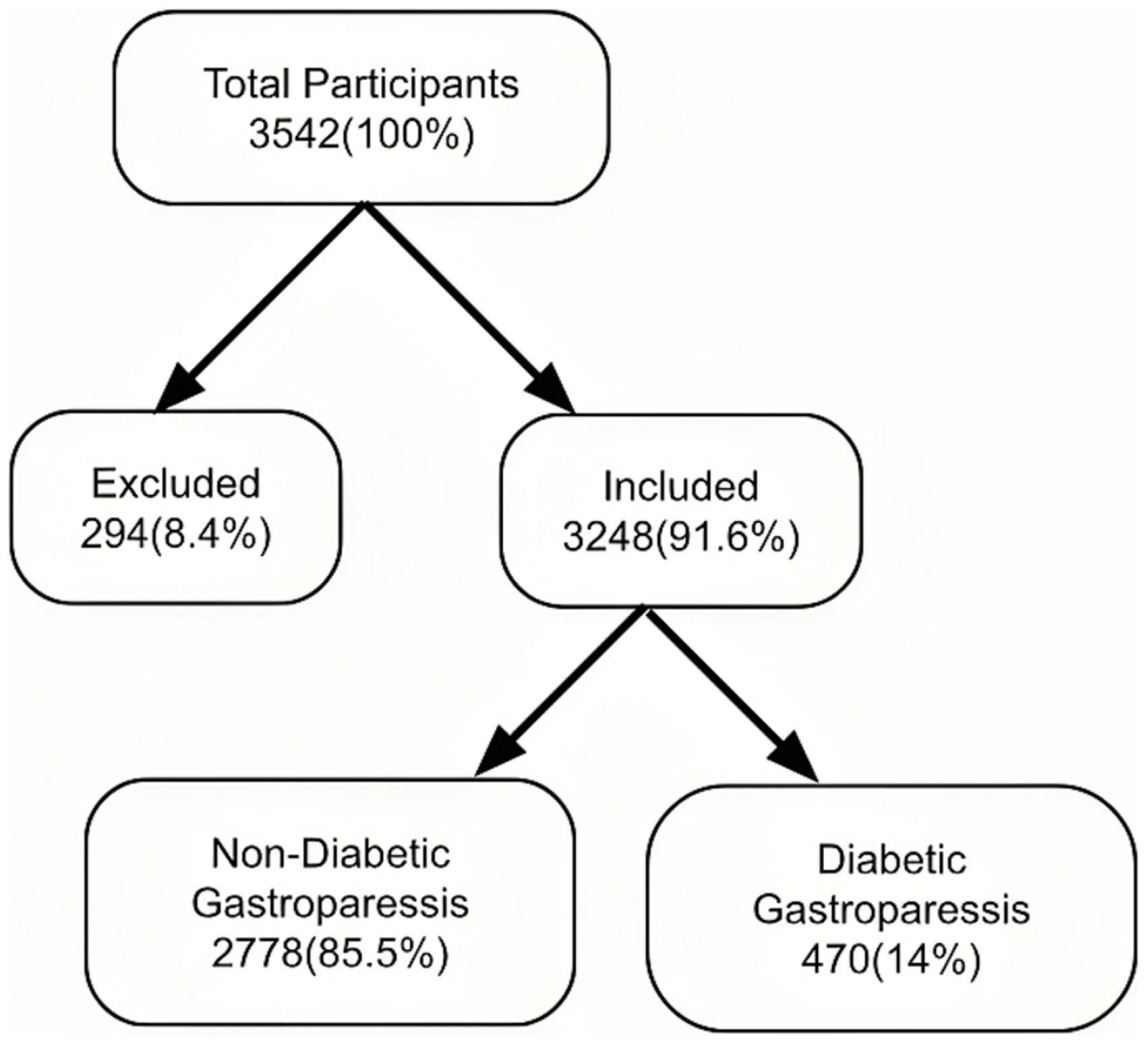
A flow diagram of the study population.

A total of 3,542 patients participated in the study and completed the questionnaire. Of these, 8.4% did not meet the inclusion criteria. Consequently, 3,248 (91.6%) were included in the analysis (Figure 1: Flow diagram). The demographic characteristics of the study participants were summarized in Table 1, which compares the DGP group with non-DGP population.

**Table 1:** Demographic characteristics of the study participants.

Demographic data	Total *N* (%)	Diabetic gastroparesis *N* (%)	Non-diabetic gastroparesis *N* (%)	*p* value
*N* (%)	3,248 (100%)	470 (14.5%)	2,778 (85.5%)	
Age (year)	61 (10 SD)	61 (12 SD)	61 (12.3 SD)	0.834
Sex				0.001
Male	1,321 (40.7%)	145 (4.5%)	1,176 (36.2%)	
Female	1927 (59.3%)	325 (10.0%)	1,602 (59.3%)	
BMI^*^(kg/m^2^)	31.1 (11.6 SD)	30.9 (6.6 SD)	31.1 (12.3 SD)	0.768
<18.5	25(0.9%)	8 (0.3%)	17 (0.6%)	
18.6–24.9	423 (14.5%)	62 (2.1%)	361 (12.4%)	
25–29.9	1,031 (35.3%)	144 (4.9%)	887 (30.4%)	
>30(obese)	1,438 (49.3%)	222 (7.6%)	1,216 (41.7%)	0.463
FBS ^**^(mg/dL)	188 (82 SD)	212 (93 SD)	184 (80 SD)	0.001
HbA1c^***^ (%)	8.36 (1.98 SD)	8.91 (2.09 SD)	8.26 (1.95 SD)	0.001
< 7	661 (23.7%)	60 (2.2%)	601 (21.5%)	
7–9	1,342 (48.1%)	186 (6.7%)	1,156 (41.4%)	
>9	787 (28.2%)	161 (5.8%)	626 (22.4%)	
Duration of diabetes (year)	12 (9 SD)	14 (9 SD)	12 (8 SD)	0.001
< 5	661 (20.4%)	82 (2.5%)	579 (17.8%)	
5–10	1,075 (33.1%)	118 (3.6%)	957 (29.5%)	
> 10	1,512 (46.6%)	270 (8.3%)	1,242 (38.2%)	
Managed with diet	1,554 (55.3%)	181 (6.4%)	1,373 (48.8%)	0.47
Uses insulin	1,132 (40.3%)	201 (7.2%)	931 (33.1%)	0.001
Uses metformin	2,434 (82.7%)	283 (10.1%)	2041 (72.6%)	0.039
Physical activity	28 (0.9%)	3 (0.1%)	25 (0.8%)	0.570

*BMI: Body Mass Index.

**FBS: Fasting Blood Sugar.

***HbA1c: Glycated hemoglobin.

### Prevalence of DGP

The prevalence of DGP in the West Bank was estimated to be 14.5%. Of these, a GCSI score indicative of a severe form of the disease was found in 10.2% of the study participants (Table 2). Patients reported that nausea (25%) and bloating (24%) were the first symptoms to occur, and the most common symptom was stomach fullness (93.6%). Approximately one-third (29%) were most bothered from the bloating compared to other symptoms.

**Table 2:** Symptoms suggestive of gastroparesis.

Symptoms	Total *N* (%)	Diabetic gastroparesis *N* (%)
Nausea	1,275 (39.3)	414 (88.1)
Retching	972 (29.9)	364 (77.4)
Vomiting	570 (17.5)	236 (50.2)
Stomach fullness	1,536 (47.3)	440 (93.6)
Early satiety	1,457 (44.9)	423 (90)
Feeling excessively full after meals	1,304 (40.1)	420 (89.4)
Loss of appetite	1,304 (40.1)	389 (82.8)
Bloating	1,517 (46.7)	406 (86.4)
Stomach or belly visibly larger	1,082 (33.3)	358 (76.2)

### Associations and risk factors of DGP

**Figure 2:**
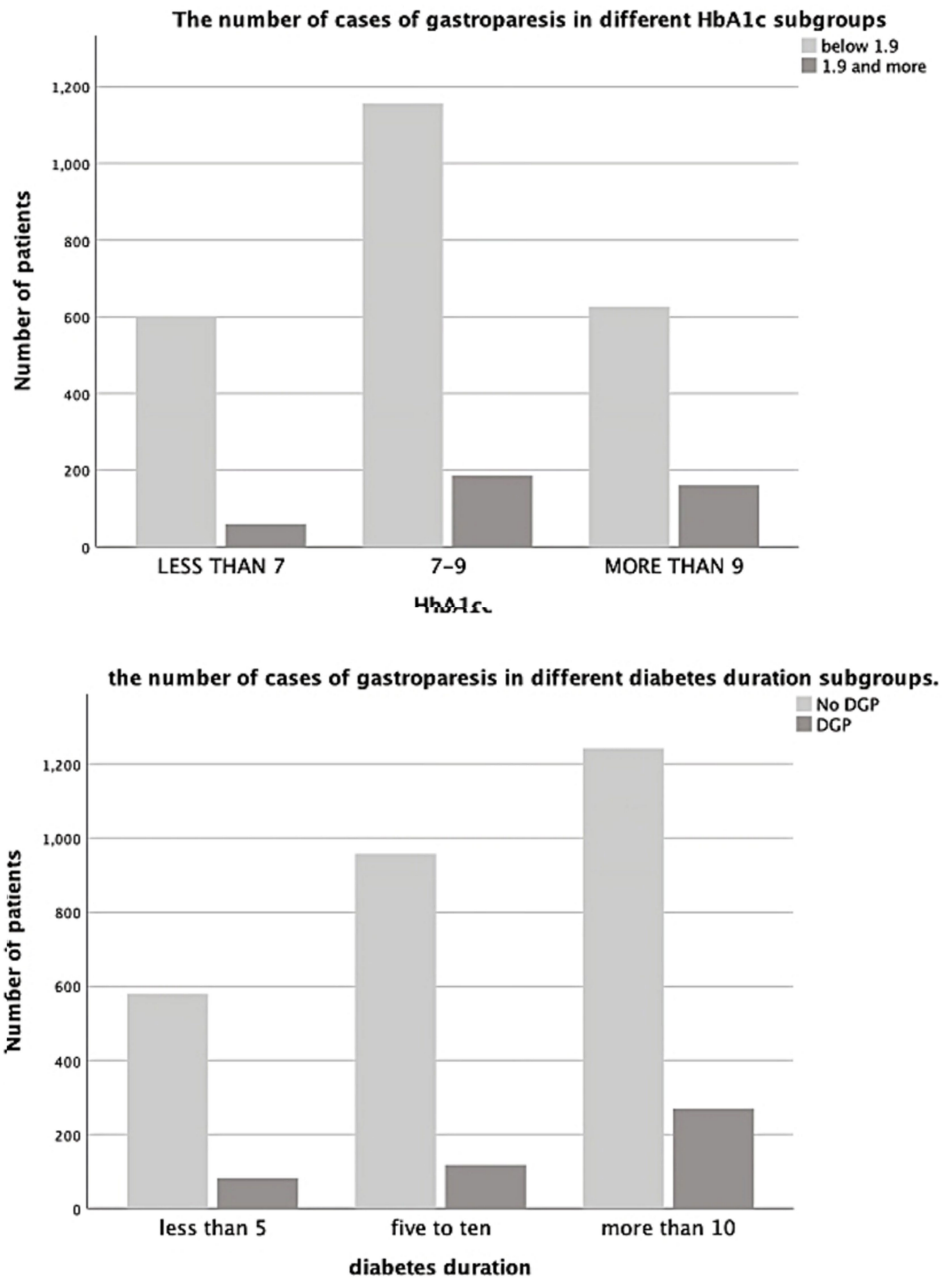
The number of case of gastroparesis in different HbA1c subgroups and in different diabetes duration subgroups.

The GCSI score showed a significant relationship with both the duration of diabetes mellitus and HbA1c (*p* = 0.0001 and *p* = 0.007). Further analysis using ANOVA test showed a significant relationship between DGP and diabetes duration >10 years (*p* = 0.0001). On the other side, no significant relationship was found between DGP and diabetes duration <10 years (Figures 1, 2).

We devised a binary regression model using the following variables: sex, age, duration of diabetes, HbA1C, level, and GCSI score. The model was significant (*p* = 0.0001) and correctly categorized 85.1% of the cases. The model revealed that patients with diabetes duration >10 years were at a higher risk (by 73%) of developing DGP (OR = 1.731; CI: 1.360–2.204; *p* = 0.0001). HbA1c > 9 significantly increased the risk of developing DGP by 127% (OR = 2.278; CI: 1.624–3.195; *p* = 0.0001). Furthermore, the model revealed that females were at 73% higher risk of developing DGP (OR = 1.734; CI: 1.368–2.199; *p* = 0.0001), with more than double the risk of developing severe DGP (GCSI score ≥ 3.5) (OR = 2.376; CI: 1.161–4.865; *p* = 0.018) (Table 3).

**Table 3:** Binary regression of symptoms suggestive of diabetic gastroparesis.

	OR	95% CI	*p* value
Age	0.999	0.987–1.010	0.840
BMI*	0.995	0.980–1.011	0.546
HbA1c**			0.001
HbA1c (7-9)	1.391	1.006–1.924	0.046
HbA1c more than 9	2.278	1.624–3.195	0.001
Female gender	1.734	1.368–2.199	0.001
Duration of more than 10 years	1.731	1.360–2.204	0.001

^*^BMI body mass index.

^**^HbA1c: Glycated hemoglobin.

In terms of disease severity, it is noteworthy that our results did not show any correlation between severity of DGP and HbA1c, DGP nor duration of diabetes.

### Under-diagnosis of DGP

Of the 470 patients with GCSI suggestive of DGP, 50.8% (239/470) sought medical advice at least once for gastroparesis complaints, 18% (85/470) were admitted to the hospital at least once due to gastroparesis symptoms, and 10.2% (48/470) were admitted in the hospital more than once. However, only 1.9% (9/470) were actually diagnosed with DGP.

## Discussion

The prevalence of diabetes-associated upper gastrointestinal symptoms has been reported to range between 5 and 12% (19). However, our study showed that the prevalence in the West Bank was higher (14.5%), thus exceeding the worldwide prevalence. This can be interpreted in the context of a higher percentage of patients with uncontrolled diabetes mellitus in the West Bank (24). Another reason that might explain this finding is the overlooked diagnosis of DGP-related symptoms. This exceeds being a local concern and has now become a global challenge (10, 25). Our study found that only almost 2% of the patients with symptoms suggestive of DGP scores were diagnosed with DGP. The underdiagnosis of gastroparesis can be attributed to several other factors: non-specific symptom presentation of gastroparesis, including nausea, bloating, and early satiety, often overlaps with other gastrointestinal disorders, such as functional dyspepsia or irritable bowel syndrome. This overlap can lead to misdiagnosis or delayed diagnosis. Second, the limited use of advanced diagnostic tools such as gastric scintigraphy, the gold standard for diagnosing delayed gastric emptying, further exacerbates this challenge. Gastric scintigraphy is underutilized due to its high cost, limited availability in resource-constrained settings, and the need for specialized equipment and expertise. Third, there is a general lack of awareness among healthcare providers regarding the presentation and prevalence of gastroparesis, particularly in patients with Type 2 Diabetes Mellitus (T2DM). This under appreciation of gastrointestinal symptoms in diabetic patients contributes to missed opportunities for early diagnosis.

Several strategies can be implemented to address these barriers. Educational initiatives to train healthcare providers on gastroparesis’ clinical presentation and diagnostic criteria could improve early recognition. Simplified and more accessible diagnostic tools, such as validated symptom questionnaires (e.g., the Gastroparesis Cardinal Symptom Index) or breath tests, should be integrated into routine care to overcome the limitations of advanced diagnostics like scintigraphy. Additionally, a multidisciplinary approach involving endocrinologists, gastroenterologists, and primary care physicians is essential to ensure comprehensive evaluation and management of patients with relevant symptoms. By addressing these gaps, it is possible to enhance the early detection and diagnosis of gastroparesis, ultimately improving patient outcomes.

In the present study, a significant correlation was found between the duration of type 2 diabetes and the GCSI score suggestive of DGP, especially in patients with diabetes mellitus ≥10 years. This was in concordance with previous studies (26–28). Recent evidence suggests that long standing diabetes mellitus correlates with gastric dysmotility, delayed gastric emptying, and upper gastrointestinal symptoms (29). This can be better understood in the context that patients with long term diabetes typically have a higher incidence of neuropathy (30). Moreover, it can be considered that DGP is a type of neuropathy associated with impaired neuronal activity of gastric function (31–34).

Our findings indicated a significant correlation between HbA1C and GCSI score, in particular above 9%. This finding aligns with what is already known about the effects of poor glycemic control and gastroparesis (26, 34–39). One study reported that hyperglycemia was associated with reduction of postprandial antral contractile activity in patients with diabetes (40). With a 2.3 times increased risk of DGP symptoms in patients with HbA1c > 9, a very similar result was reported by Sogabe et al. (41) comparing gastric motility in DGP patients before and after gaining glycemic control and found that there was an improvement in gastric motility and reduction in symptoms after achieving near-optimal glycemic control (42). A Japanese study concluded that gastric myoelectrical activity was partially regulated by glycemic control and autonomic nerve function (43). Thus, any change in this myoelectrical activity can be reversed by controlling plasma glucose. This accentuates the role of glycemic control in the management of DGP.

Female predominance was observed in our study in terms of increased risk of developing DGP. Furthermore, they were more likely to develop severe forms of the disease compared to males. Literature reveals that the prevalence of gastroparesis in female patients can be as high as 88% (44), with female to male ratio of 4:1 (45, 46). Studies have proposed different mechanisms to provide a plausible explanation. It is believed that female sex hormones play a crucial role in gastric motility (47–52). New evidence has shown that estrogen and progesterone receptors found in the gastrointestinal (GI) tract influences GI motility (53, 54). Studies which assessed gastric motility during the menstrual cycle reported that elevated estrogen and progesterone levels during the luteal phase slowed gastric emptying (55–57). Gender-related physiological differences also exist. Females have decreased antral contractility, enteric transmission, and visceral hypersensitivity compared to males (49, 54, 58, 59).

We found that most patients were on metformin (60.2%). Oral anti-diabetic medications such as metformin and alpha-glucosidase inhibitors can cause GI symptoms (pain, nausea, bloating). Although there is a well-established relationship between metformin and gastroparesis (60), its pharmacokinetic effects are not well understood. It has been suggested to delay gastric emptying due to DPP-4 inhibition (61). Further studies are needed to assess the effect of metformin on patients’ gastric emptying. However, the key point is that when metformin should be stopped to improve the patients´ symptoms (62, 63). A crucial aspect highlighted in the study by Alam et al. [62] is the timing of metformin discontinuation to improve symptoms in diabetic gastroparesis patients. The study emphasizes the importance of identifying when cessation of the medication may be beneficial for symptom management-- However, the key point is determining when metformin should be discontinued to alleviate the patient’s symptoms.

One limitation of our study was the unavailability of scintigraphy, which is the gold standard for diagnosing DGP (6). Instead, we used the GCSI to classify patients with DGP, a tool that has been validated as reliable and effective for measuring the severity of gastroparesis symptoms (21). Additionally, while our study provides valuable insights into the prevalence and associated factors of DGP symptoms, it did not stratify or analyze the effects of specific medication types on these symptoms. This limitation is inherent to the cross-sectional design and the lack of detailed data on individual medication regimens. Notably, most patients in our study settings were prescribed metformin as part of their diabetes management plan. Although medications such as GLP-1 receptor agonists and SGLT2 inhibitors are included in the Palestinian Essential Medication List, their use in governmental healthcare centers is limited by cost constraints and availability. This likely reduced variability in medication-induced gastrointestinal symptoms among the participants. Lastly, the study timetable was interrupted by the Coronavirus (23) disease (COVID-19) pandemic, which delayed the data collection phase.

In conclusion, DGP is an underdiagnosed condition. Steps have been taken to improve this aspect, but there is still a long way to go regarding diagnosis and management that would translate into clinical practice to optimize healthcare provided for DGP patients. Addressing the recognition and management of diabetic gastroparesis (DGP) requires a multifaceted approach involving both clinical and systemic improvements. Clinicians should routinely utilize validated screening tools such as the Gastroparesis Cardinal Symptom Index (GCSI) to identify DGP symptoms, particularly in resource-limited settings. Screening should prioritize high-risk patients, such as those with diabetes for ≥10 years, females, or those with poorly controlled HbA1c levels. Comprehensive patient evaluation is crucial, with a focus on differentiating DGP symptoms from medication side effects, and early referral to a multidisciplinary team can significantly enhance patient outcomes. Ensuring optimal glycemic control is vital in preventing and managing DGP symptoms, with individualized dietary modifications, pharmacologic interventions, and attention to modifiable factors like hydration and nutrition. On a broader scale, policymakers should work to improve healthcare infrastructure, providing better access to tools like the GCSI and advanced methods such as gastric scintigraphy, particularly in resource-constrained areas. Training healthcare providers through continuing medical education and launching awareness campaigns for both professionals and patients can significantly raise awareness about the importance of diagnosing and managing gastrointestinal complications in diabetes. Moreover, integrating DGP care into national diabetes guidelines and facilitating early diagnosis and referral pathways will streamline care and improve outcomes. As part of ongoing efforts to enhance diagnosis and awareness, we collaborated with the Palestinian Society of Gastroenterology, sharing the translated Arabic version of the GCSI to promote its adoption in local hospitals and clinics. This initiative aims to increase awareness among healthcare providers and patients, marking a key step toward improving DGP care at a systemic level. Future research should focus on the impact of diabetes medications on gastrointestinal symptoms and explore various treatment modalities for DGP in diabetic populations to reveal the hidden part of the diabetic gastroparesis iceberg. Additionally, fostering collaboration between healthcare institutions and researchers will be essential to gathering national and regional data, ultimately informing evidence-based policy decisions.

## Collaborators

Lana Khatib, Lema Jaber, Afnan Radaydeh, Bayan Saada, Äsmää Hasan Zäbädiah, Nurhan Al-hroub, Zeina Ihab Ghazaleh, Zain Saqfalhait, Mayada Saad Taha, Mayas Naim, Manwa Atef, Samar Yaqoub, Rawan Abu Sa’da, Qais Nasalla, Hamdoon Abu-Arish, Areej Marwan, Eliana Masri, Amani Ahmed, Siba Alrojoub.

## Data Availability

The original contributions presented in the study are included in the article/supplementary material, further inquiries can be directed to the corresponding author.
